# Angiotensin-(1-7)-Mediated Signaling in Cardiomyocytes

**DOI:** 10.1155/2012/493129

**Published:** 2012-03-04

**Authors:** Enéas R. M. Gomes, Robson A. S. Santos, Silvia Guatimosim

**Affiliations:** ^1^Department of Physiology and Biophysics, Institute of Biological Sciences, Federal University of Minas Gerais, 31270-901 Belo Horizonte, MG, Brazil; ^2^National Institute of Science and Technology in Nanobiopharmaceutics, Institute of Biological Sciences, Federal University of Minas Gerais, 31270-901 Belo Horizonte, MG, Brazil

## Abstract

The Renin-Angiotensin System (RAS) acts at multiple targets and has its synthesis machinery present in different tissues, including the heart. Actually, it is well known that besides Ang II, the RAS has other active peptides. Of particular interest is the heptapeptide Ang-(1-7) that has been shown to exert cardioprotective effects. In this way, great compilations about Ang-(1-7) actions in the heart have been presented in the literature. However, much less information is available concerning the Ang-(1-7) actions directly in cardiomyocytes. In this paper, we show the actual knowledge about Ang-(1-7)-mediated signaling in cardiac cells more specifically we provide a brief overview of ACE2/Ang-(1-7)/Mas axis; and highlight the discoveries made in cardiomyocyte physiology through the use of genetic approaches. Finally, we discuss the protective signaling induced by Ang-(1-7) in cardiomyocytes and point molecular determinants of these effects.

## 1. Introduction

Arterial hypertension is an important cardiovascular risk factor and contributes to the development of cardiovascular event. Despite the substantial advances in antihypertensive drug therapy, the number of patients with uncontrolled hypertension remains high around the world [1].

The renin-angiotensin system (RAS) is an important classical player that directly contributes to the development and maintenance of essential hypertension [2]. This system is classically known as a hormonal system, involved in salt and water regulation and blood pressure control. Angiotensin (Ang) II, one of the main components of the RAS, exerts its biological effects by binding with high affinity to two distinct subtypes of receptor, the angiotensin II type 1 receptor (AT_1_R) and the angiotensin II type 2 receptor (AT_2_R) [3, 4]. Under physiological and pathological states, it is recognized that AT_1_R plays a critical role in Ang II-mediated actions in the cardiovascular system [3, 4]. On the other hand, a large body of evidence suggests that AT_2_R antagonizes the effects of AT_1_R preventing, between other effects, its hypertrophic and angiogenic effects [5, 6].

 Although Ang II is the major effector of this system, several other peptides are now recognized as being biologically important. Of particular importance is the heptapeptide Ang-(1-7) that decades ago emerged as a new metabolite of the RAS. Ang-(1-7) was initially detected as an Ang I metabolite in canine brain homogenates [7]. This discovery led to the later demonstration of its action in releasing vasopressin from hypophyseal-hypothalamic explants [8] and in counteracting the pressor and baroreflex effects of Ang II [9–11]. Later on, Ang-(1-7) was finally recognized as a putative biologically active component of the RAS [12–14]. Since then, the physiological actions of Ang-(1-7) have been extensively investigated. The heart is an important target for Ang-(1-7), which exerts direct effects on cardiomyocytes. The following sections focus on the cellular mechanism and signaling pathways involved in Ang-(1-7) actions in the cardiac cell, with particular emphasis on recent discoveries made through the use of genetic approaches.

## 2. The Angiotensin-Converting Enzyme2/Angiotensin-(1-7)/Mas Axis

**Figure 1:**
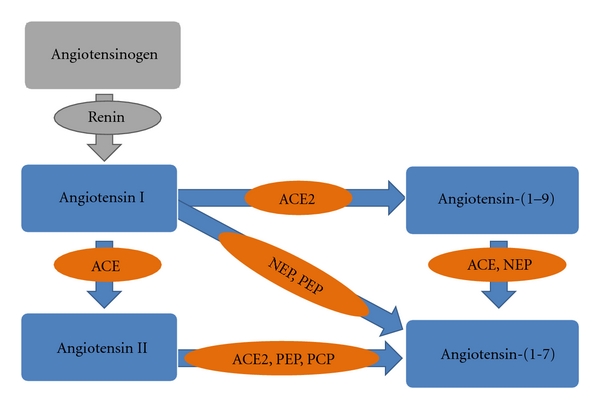
Schematic representation of the enzymatic pathways involved in the generation of Ang-(1-7). Ang-(1-7) can be formed by at least three different pathways: directly from Ang I by NEP and PEP, by hydrolysis of Ang II by ACE2, PEP, and PCP, and finally by hydrolysis of Ang-(1-9) by ACE and NEP. ACE, ACE2, and NEP are found in cardiomyocytes.

Ang-(1-7) can be formed directly from Ang I or Ang II and indirectly from Ang I having as an intermediate step the formation of Ang-(1-9) [14, 15]. Angiotensin-converting enzyme (ACE) 2, as well as prolylcarboxypeptidase (PCP) and prolylendopeptidase (PEP), can generate Ang-(1-7) directly from Ang II. Apparently, the principal enzyme and pathway involved in the Ang-(1-7) generation is ACE2 through the hydrolysis of Ang II [16, 17]. However, Campbell et al. [18] have suggested that PEP is the main enzyme responsible for generating Ang-(1-7) from Ang II in human coronary vessels. In addition, it appears that the generation of Ang-(1-7) involving an intermediate step, including the hydrolysis of Ang I to Ang-(1-9), is less important [19]. Ang-(1-7) is also produced directly from Ang I by neutral endopeptidase (NEP) and PEP through hydrolysis of the Pro7-Phe8 bond [19, 20]. A schematic representation of the enzymatic pathways involved in the generation of Ang-(1-7) is presented in Figure 1.

In cardiomyocytes the expression of ACE2, the main enzyme involved in Ang-(1-7) formation, was already demonstrated [21, 22], as well as NEP [23]. In addition, ACE mRNA has been consistently demonstrated in cardiac cells [24, 25]. However, the ACE mRNA levels were not supported by protein measurements in human hearts. No immunoreactivity for ACE was found in ventricular myocytes from human control hearts, with ACE detected only in cardiomyocytes from hearts after myocardial infarction [26]. Nevertheless, ACE has been demonstrated in cardiomyocytes from rats and mice by enzyme-kinetic and immunohistochemical methods [27]. The presence in the cardiomyocyte of these specific enzymes indicates that Ang-(1-7) and some of RAS components can be locally synthesized in the heart. It remains to be determined which components are produced locally and in which conditions this production is activated.

The identification of ACE2 as an important Ang-(1-7)-forming enzyme [28, 29], and of Mas as a G protein-coupled receptor for Ang-(1-7) [30], contributed to establish Ang-(1-7) as a biologically active component of the RAS. In 2005, Ferreira and Santos advanced the hypothesis that ACE2, Ang-(1-7), and Mas could be considered as components of a novel axis of the RAS, the so-called ACE2/Ang-(1-7)/Mas axis [31]. For the heart, this concept is now quite well accepted in the literature [13, 32–35]. But, at present, only limited information is available regarding the direct effects of ACE2/Ang-(1-7)/Mas axis activation for cardiomyocyte function during physiological as well as in pathological conditions. In addition to ACE2, the presence of Mas has been demonstrated in cardiomyocytes from different species [21, 22, 36, 37], including humans [38].

**Figure 2:**
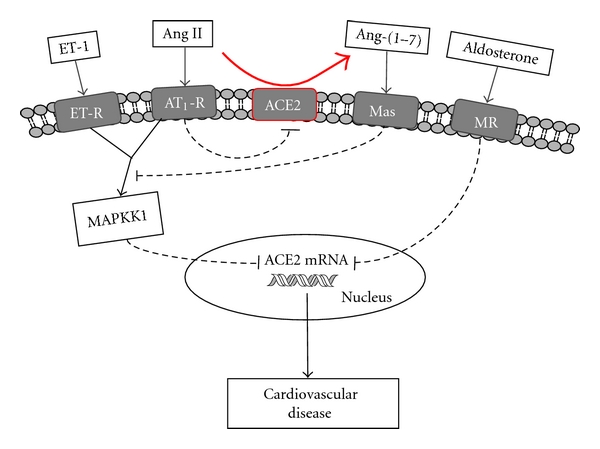
Schematic illustration of ACE2 modulation in cardiomyocytes by different molecules. Ang II, and ET-1 through MAPKK1 activation, and aldosterone lead to ACE2 mRNA downregulation. To date no reported effects of Ang-(1-7) on ACE2 levels have been demonstrated, although this peptide is capable of antagonizing Ang II and ET-1 effects on ACE2 mRNA. Conditions that favor ACE2 downregulation would likely lead to more deleterious effects during cardiovascular disease development.**→**= activation; - -⊣= inhibition; ET-1 = endothelin; Ang II = angiotensin II; Ang-(1-7) = angiotensin-(1-7); ET-R = endothelin receptor; AT_1_-R = AT_1_ receptor; Mas = Mas receptor; MR = mineralocorticoid receptor; MAPKK1 = mitogen-activated protein kinase kinase 1.

A variety of vasoactive peptides and hormones can regulate ACE2 mRNA levels in cardiomyocytes. Modulation of ACE2 mRNA levels by aldosterone has been demonstrated in neonatal cardiomyocytes treated with this mineralocorticoid [22]. Accordingly, aldosterone decreased ACE2 mRNA levels in these cells, an effect apparently mediated by the mineralocorticoid receptor. In the same study, ACE2 mRNA modulation was not affected by Ang II treatment, suggesting that ACE2 mRNA expression is under differential modulation by endocrine molecules in cardiomyocytes. Considering the Ang II actions on ACE2 mRNA levels, opposing results were obtained by Gallagher et al. [21], who found a decrease in ACE2 activity and downregulation of its mRNA by Ang II. Importantly, this effect was mediated by AT_1_R and blocked by inhibitors of mitogen-activated protein kinase kinase 1 (MAPKK1). Considering that differences in experimental conditions can explain the contrasting results regarding Ang II modulation of ACE2, further investigation will be necessary to elucidate the specific mechanism involved in ACE2 downregulation in cardiomyocytes. Endothelin-1 (ET-1) also significantly reduced myocyte ACE2 mRNA via MAPKK1 activation [21]. Apparently Ang-(1-7) has no direct effect on ACE2 mRNA regulation, although this peptide, through Mas receptor, blocked the Ang II and ET-1 mediated downregulation of ACE2 expression [21]. Collectively, these results indicate that ACE2 expression in cardiomyocytes is tightly regulated by important modulators of cardiovascular system, highlighting its importance in cardiac disease establishment and progression. Since ACE2 converts Ang II to Ang-(1-7), it is plausible that ACE2 downregulation by Ang II serves as a mechanism to favor Ang II-mediated responses, by preventing its degradation to Ang-(1-7). Thus, conditions favoring excess Ang II generation and reduced Ang II breakdown would likely lead to more deleterious effects on the heart.  Figure 2 summarizes the information regarding the modulation of ACE2 expression in cardiomyocytes.

## 3. Protective Signaling Induced by ACE2/Ang-(1-7)/Mas Axis in Cardiomyocytes

**Figure 3:**
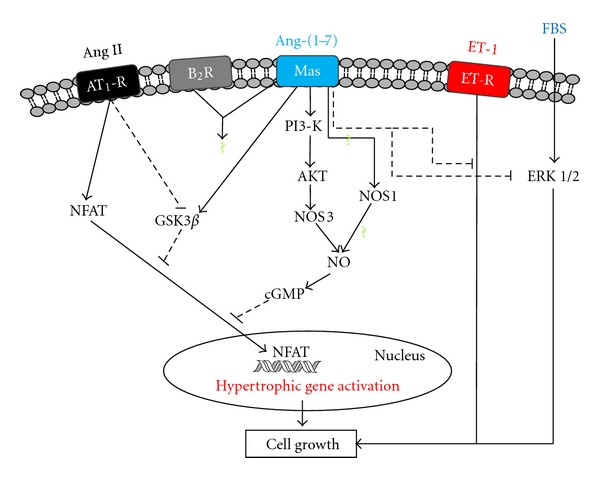
Ang-(1-7) signaling in cardiomyocytes. Ang-(1-7) activates the PI3-K/AKT/NOS3 pathway leading to NO generation and cGMP production. Activation of this pathway culminates with inhibition of Ang II-induced NFAT translocation. Preliminary evidence suggests that NOS1 expression may be modulated by Ang-(1-7). Ang-(1-7) also inhibits ET-1 and FBS activation of cell growth. The consequences of Ang-(1-7)/Mas and B_2_R cross-talk for cardiomyocyte function are still unknown (?). **→**= activation; - -⊣= inhibition; ET-1 = endothelin; Ang II = angiotensin II; Ang-(1-7) = angiotensin-(1-7); ET-R = endothelin receptor; AT_1_-R = AT_1_ receptor; Mas = Mas receptor; B_2_R = bradykinin receptor type 2; FBS = fetal bovine serum; NFAT = nuclear factor of activated T cells; GSK3*β* = glycogen synthase kinase 3*β*; PI3-K = phosphatidylinositol 3-kinase; AKT = protein kinase B; NOS3 = nitric oxide synthase 3; NOS1 = nitric oxide synthase 1; NO = nitric oxide; cGMP = cyclic guanosine monophosphate; ERK 1/2 = extracellular signal regulated kinase 1/2. The question marks denote areas in which the current state of knowledge is still preliminary.

 In the past two decades, since the detection of the Ang-(1-7) as a product of the metabolism of Ang I, the physiological actions of Ang-(1-7) have been extensively investigated, and Ang-(1-7) was finally recognized as a putative biologically active component of the RAS [12–14]. However, besides this great advance in the understanding of Ang-(1-7) actions, especially in the heart, only a few reports have explored the Ang-(1-7) actions directly in cardiomyocytes. This section will focus on signaling pathways and molecular determinants of Ang-(1-7) signaling in cardiomyocytes. This will be accomplished by highlighting the following effects: (1) antihypertrophic, (2) anti-inflammatory, and (3) antioxidative. In addition, we summarize current knowledge regarding Ang-(1-7) modulation of Ca^2+^ handling in cardiomyocytes. Initial studies were performed by Tallant et al. [39], who confirmed the presence of the Mas receptor in neonatal cardiomyocytes and showed a direct effect of Ang-(1-7) in these cells, by preventing cell growth, through inhibition of the MAPK ERK1/2 activity. Later on, it was demonstrated the presence of Mas receptor in adult ventricular myocytes [40]. Continuing the exploration of Ang-(1-7) actions and pathways in cardiomyocytes, some information coming from different types of cell supported the next steps in the understanding of the signaling molecules involved in the Ang-(1-7) effects. Sampaio et al. [41] showed that in endothelial cells Ang-(1-7) was able to generate nitric oxide (NO). In the same way, Dias-Peixoto et al. [36] demonstrated that Ang-(1-7) was able to activate the phosphatidylinositol 3-kinase (PI3-K)-protein kinase B (Akt)-pathway, resulting in nitric oxide synthase (NOS) 3 activation and NO generation in adult ventricular cardiomyocytes. It should be noted that cardiomyocytes express distinct subtypes of PI3-K, and some of them are activated by Ang II [42, 43]. Therefore, is of particular importance to investigate which specific pools of PI3-K are regulated by Ang II and Ang-(1-7). In addition, Dias-Peixoto et al. [36] have shown that expression levels of proteins involved in the NOS3 macromolecular complex, such as caveolin-3, heat shock protein (HSP)-90, and protein kinase B (AKT), were altered in ventricular myocytes from *Mas^−/−^* (Mas knockout) mice, indicating an important relationship between NOS3 activity and Ang-(1-7)/Mas axis. Initial investigation into the cellular mechanisms underlying protective effects of Ang-(1-7) against Ang II signaling was recently performed by our laboratory. Gomes et al. [37] have demonstrated, in cardiomyocytes, that Ang-(1-7) prevention of Ang II-induced pathological remodeling is mediated by NO/cGMP (cyclic guanosine monophosphate) pathway. This result identifies a role of NO as mediator of Ang-(1-7) beneficial effects and extends the concept that cGMP is another key molecule in this signaling pathway. In addition, this study showed that transgenic rats presenting increased Ang-(1-7) plasmatic levels have higher levels of NOS1 in ventricular cardiomyocytes, showing that besides NOS3, NOS1 shall be involved in NO generation elicited by Ang-(1-7). Ang-(1-7) also modulated the activity of the transcription factor NFAT (nuclear factor of activated T cells), preventing its translocation to the nucleus, and the activation of hypertrophic gene program by Ang II [37]. Stimulated by calcium signals, NFAT is translocated to the nucleus where it can regulate hypertrophic genes. In cardiomyocytes, NFAT nuclear localization is tightly controlled at multiple levels [44–46]. Glycogen synthase kinase 3*β* (GSK3*β*), in particular, is considered a potent inhibitor of this pathway downstream of calcineurin. In the nucleus, GSK3*β* phosphorylates NFAT, thereby promoting its nuclear export [44]. Moreover, GSK3*β* has been shown to regulate hypertrophy development by restraining gene expression [47]. Gomes et al. [37] have shown that Ang-(1-7) modulates the activity of GSK3-*β*, by preventing its inactivation by Ang II. The modulation of these two proteins, NFAT and GSK3- *β*, supports the anti-hypertrophic effect of Ang-(1-7) observed in the heart [37] and in cardiomyocytes [37, 39]. Corroborating these findings Flores-Muñoz et al. [48] reported that Ang-(1-7) was able to block the increase in cell size induced by Ang II in H9c2 cardiomyocytes. These effects were mediated by Mas receptor, since Mas antagonist A779 efficiently blocked the antihypertrophic effects of Ang-(1-7). Importantly, these authors have also shown that Ang-(1-7) anti-hypertrophic activity was inhibited in the presence of the bradykinin type 2 receptor (B_2_R) antagonist, HOE140, suggesting a cross-talk between Mas and B_2_R in response to Ang-(1-7).  Figure 3 shows recent data about Ang-(1-7) signaling and cross-talk in cardiomyocytes.

Recently, Qi et al. have provided evidence for an anti-inflammatory role of angiotensin-(1-7) at the cardiomyocyte level [49]. By using neonatal cardiomyocyte culture, the authors demonstrated that protective effects of Ang-(1-7) against hypoxia-induced cell death were mediated, at least in part, through modulation of cytokine production. This beneficial effect was associated with decreased expression of inflammatory cytokines such as tumor necrosis factor-*α* (TNF-*α*) and interleukin-6 (IL-6) and increased gene expression of ACE2, bradykinin type 2 receptor, and interleukin-10 (IL-10). Taken together, these data show that Ang-(1-7) regulates cytokine responses, which could contribute to its cardioprotective effects.

Considering the critical role of Ca^2+^ ions for cardiomyocyte contraction [50], some studies have addressed whether Ang-(1-7) modulates Ca^2+^ handling in ventricular cardiomyocytes. Recent work by our group has shown that acute Ang-(1-7) treatment does not significantly alter Ca^2+^ transient amplitude or kinetics of decay [36]. We extended these findings to *in vivo* conditions and showed that cardiomyocytes from transgenic (TG) rats with chronic elevated plasmatic Ang-(1-7) do not show alteration in cytosolic Ca^2+^ transient parameters [37]. Interestingly, cardiomyocytes from mice with genetic ablation of Ang-(1-7) Mas receptor (*Mas^−/−^*) presented a Ca^2+^ signaling dysfunction represented by a smaller peak Ca^2+^ transient and slower Ca^2+^ uptake. This Ca^2+^ signaling dysfunction was accompanied by decreased protein levels of the sarcoplasmic/endoplasmic reticulum Ca^2+^ ATPase 2 (SERCA2) [36]. SERCA2 is responsible for Ca^2+^ reuptake by the sarcoplasmic reticulum (SR), thereby setting SR Ca^2+^ load, which is an important determinant of Ca^2+^ release in cardiomyocytes [50]. The reduction in the Ca^2+^ transient is consistent with the depression of contractility that was previously observed in *Mas* ^−/−^ hearts [40]. This finding was particularly important since it suggested that the Ang-(1-7)/Mas axis is critical for long-term maintenance of normal Ca^2+^ handling in the cardiac cell. However, there was still the possibility that the alterations in Ca^2+^ handling found in *Mas^−/−^* cardiomyocytes were secondary to the cardiac dysfunction observed in these hearts.

 Adding further complexity to the understanding of Ang-(1-7)/Mas modulation of cardiomyocyte Ca^2+^  signaling, it was also shown that cardiomyocytes from TG rats with cardiac specific overexpression of Ang-(1-7) presented higher Ca^2+^ transient amplitude, faster Ca^2+^ uptake, and increased levels of SERCA2 [51], suggesting that chronic local increase of Ang-(1-7) in the heart was associated to enhanced Ca^2+^ handling. Are these changes in Ca^2+^ handling a direct consequence of local Ang-(1-7) increase in the heart? These findings contrasted with the lack of effect on Ca^2+^ signaling found in cardiomyocytes from TG rats with chronic elevated plasmatic Ang-(1-7) levels. As it stands, the relationship between Ang-(1-7) and Ca^2+^ signaling is more complex than one may have anticipated. It is also plausible, that Ang-(1-7) effects on Ca^2+^ handling observed in an *in vivo* model of chronic Ang-(1-7) overexpression in the heart are consequences of long-term changes in expression levels of Ca^2+^ handling proteins. Future studies are needed to demonstrate whether Ang-(1-7) prevents Ca^2+^ signaling dysfunction in ventricular myocytes from animal models of heart failure.

**Figure 4:**
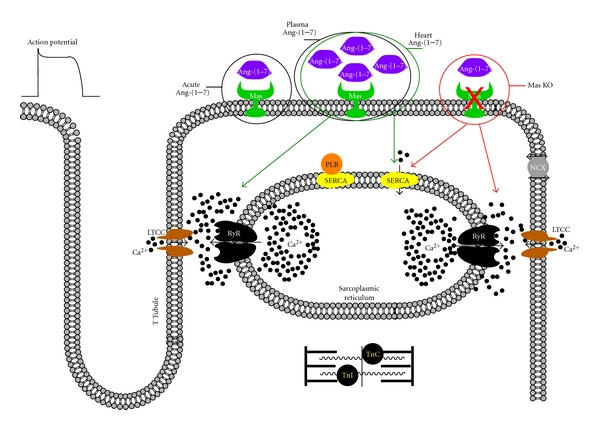
Summary of current knowledge regarding Ang-(1-7) modulation of Ca^2+^ handling in ventricular myocytes and the underlying mechanisms. Acute treatment of cardiomyocytes with Ang-(1-7) apparently has no direct effect on Ca^2+^ handling (black circle, left). Lack of effect on Ca^2+^ levels was also observed in cardiomyocytes from TG rats with increased circulating levels of Ang-(1-7) (black circle, middle). In contrast, Ang-(1-7) signaling disruption through Mas genetic ablation (Mas KO) leads to Ca^2+^ dysfunction (red circle, right). Cardiomyocytes from Mas KO mice present reduced SERCA expression levels and Ca^2+^ transients (red arrows). Cardiac specific overexpression of Ang-(1-7) enhances Ca^2+^ release and SERCA levels in ventricular myocytes (green circle and arrows). Data regarding Ang-(1-7) modulation of some other key proteins involved in Ca^2+^ handling in ventricular myocytes, such as PLN, NCX, TnI, and TnC, are still missing. Black filled circles = calcium ions; LTCC = L-type Ca^2+^channels; Mas = Mas receptor; NCX = Na^+^/Ca^2+^ exchanger; PLB = phospholamban; RyR = ryanodine receptor; TnC = troponin C; TnI = troponin I.

NO has been attributed as a key mediator of Ang-(1-7) effects on different cell types, including cardiomyocytes, and it is known to interact with proteins involved in Ca^2+^ handling and regulate cardiac contractility. The question remains whether long-term Ang-(1-7) effects on Ca^2+^ handling are mediated by NO or other signaling molecules in cardiomyocytes. In this way, the regulation of some key proteins involved in cardiomyocyte Ca^2+^ handling, such as ryanodine receptor (RyR), phospholamban (PLN), Na^+^/Ca^2+^ exchanger (NCX), and troponins by Ang-(1-7) must be investigated in order to provide a deeper understanding of Ang-(1-7) actions on Ca^2+^ signaling. A summary of current knowledge regarding Ang-(1-7)/Mas modulation of cardiomyocyte Ca^2+^ signaling is shown in Figure 4.

Evidence for a direct role of ACE2/Ang-(1-7)/Mas axis against oxidative stress in cardiomyocytes was also obtained [52]. Experiments on adult ventricular myocytes demonstrated that Ang II-mediated superoxide generation and extracellular signal-regulated kinase 1/2 (ERK 1/2) activation were inhibited by recombinant ACE2 (rhACE2). Importantly, these effects were mediated by Ang-(1-7), since preincubation with the Mas receptor peptide antagonist, D-Ala7-Ang-(1-7), largely prevented rhACE2 suppression of Ang II-induced responses in cardiomyocytes. These *in vitro* findings correlated with *in vivo* data showing that treatment with rhACE2 prevented Ang II–induced hypertrophy and myocardial fibrosis. Thus, these findings give further support to the fact that enhanced Ang-(1-7) signaling at the cardiomyocyte level prevents Ang II pathological effects and highlight Ang-(1-7) anti-oxidative actions on cardiomyocytes.

Contrasting to the view of a protective role of ACE2/Ang-(1-7)/Mas axis some reports have shown hypertrophic effects of ACE2 and Mas overexpression in cardiomyocytes. Masson et al. [53] reported that adenoviral-mediated gene transfer of ACE2 in rabbit cardiomyocytes leads to cellular hypertrophy. In the same study, *in vivo* ACE2 overexpression in the myocardium of stroke-prone spontaneously hypertensive rats resulted in profound cardiac dysfunction. The authors have argued that the detrimental effects of ACE2 overexpression were possibly due to higher amounts of protein expressed using this approach. Using similar overexpression strategy, neonatal rat cardiomyocytes were infected with adenovirus encoding the human Mas receptor. Intriguingly, overexpression of Mas induced a significant increase in IP3 accumulation and cellular hypertrophy. These responses were due to enhanced Gq-mediated signaling via Mas receptor [38]. Whether this response is a consequence of exacerbated Mas signaling or is a result of promiscuous signaling activation caused, for example, by hetero-dimerization, it is a topic that needs further clarification. Therefore, understanding the role of “physiological” versus “supraphysiological” levels of ACE2 and Mas, its downstream signaling pathways and their functional outcomes are crucial for clarifying the role of ACE2/Ang-(1-7)/Mas axis for cardiomyocyte function.

## 4. Conclusions and Perspectives

 In cardiomyocytes, the actual knowledge of Ang-(1-7) protective effects was mainly focused on the modulation of Ang II signaling with emphasis on anti-hypertrophic actions. This effect was dependent on Mas, indicating that important cardioprotective aspects of Ang-(1-7) signaling are mediated through Mas receptor, and involved NO and cGMP generation. It remains to be elucidated whether antioxidative and anti-inflammatory responses of Ang-(1-7) also depend on NO/cGMP production. A direct acute effect of Ang-(1-7) on Ca^2+^ signaling in cardiomyocytes seems unlikely. However, there still are many ways by which Ang-(1-7) may regulate Ca^2+^ signaling in ventricular myocytes. Chronic increase in local Ang-(1-7) levels could be a mechanism by which Ang-(1-7) enhances Ca^2+^ handling, as observed in cardiomyocytes from transgenic rats with cardiac specific overexpression of Ang-(1-7). To understand how Ang-(1-7) regulates Ca^2+^ handling in ventricular myocytes is of fundamental importance in light of Ang-(1-7) therapeutic potential in several disease conditions. The literature shows a tight control of ACE2 synthesis and activity in cardiomyocytes. How this enzyme expression is modulated, the signaling pathways involved in this regulation, and whether this occurs *in vivo* are questions that remain to be answered, considering the pivotal role of ACE2 as modulator of Ang II/Ang-(1-7) levels. The actions of Ang-(1-7) on cardiomyocytes are just beginning to unravel, dissecting the signaling pathways, and the conditions under which Ang-(1-7) signaling is turned on will be a major issue to be addressed in the future.
